# Translating knowledge into action for child obesity treatment in partnership with Parks and Recreation: study protocol for a hybrid type II trial

**DOI:** 10.1186/s13012-023-01264-5

**Published:** 2023-02-24

**Authors:** Cody D. Neshteruk, Asheley C. Skinner, Julie Counts, Emily M. D’Agostino, Leah Frerichs, Janna Howard, Mary Story, Sarah C. Armstrong

**Affiliations:** 1 Department of Population Health Sciences, Duke University School of Medicine, 215 Morris Street, Suite 210, Durham, NC 27701 USA; 2 Duke Clinical Research Institute, Durham, NC USA; 3 Duke Molecular Physiology Institute, Durham, NC USA; 4 Department of Orthopaedic Surgery, Duke University School of Medicine, Durham, NC USA; 5 Department of Health Policy and Management, University of North Carolina at Chapel Hill, Chapel Hill, NC USA; 6 Department of Pediatrics, Duke University School of Medicine, Durham, NC USA; 7 Department of Family Medicine and Community Health, Duke University School of Medicine, Durham, NC USA; 8 Duke Global Health Institute, Duke University School of Medicine, Durham, NC USA

**Keywords:** Pediatrics, Obesity treatment, Implementation science, Dissemination, Mixed methods, Protocol, Physical activity, Exercise

## Abstract

**Background:**

Safe and effective treatment exists for childhood obesity, but treatment recommendations have largely not been translated into practice, particularly among racial and ethnic minorities and low-wealth populations. A key gap is meeting the recommended treatment of ≥26 h of lifestyle modification over 6–12 months. Fit Together is an effective treatment model that meets these recommendations by integrating healthcare and community resources. Pediatric providers screen children for obesity, deliver counseling, and treat co-morbidities, while Parks and Recreation partners provide recreation space for a community nutrition and physical activity program.

**Methods:**

This study will use a hybrid type II implementation-effectiveness design to evaluate the effectiveness of an online implementation platform (the Playbook) for delivering Fit Together. Clinical and community partners in two North Carolina communities will implement Fit Together, using the Playbook, an implementation package designed to facilitate new partnerships, guide training activities, and provide curricular materials needed to implement Fit Together. An interrupted time series design anchored in the Process Redesign Framework will be used to evaluate implementation and effectiveness outcomes in intervention sites. Implementation measures include semi-structured interviews with partners, before and after the implementation of Fit Together, and quantitative measures assessing several constructs within the Process Redesign Framework. The participants will be children 6–11 years old with obesity and their families (*n*=400). Effectiveness outcomes include a change in child body mass index and physical activity from baseline to 6 and 12 months, as compared with children receiving usual care. Findings will be used to inform the design of a dissemination strategy guided by the PCORI Dissemination Framework.

**Discussion:**

This project addresses the knowledge-to-action gap by developing evidence-based implementation tools that allow clinicians and communities to deliver effective pediatric obesity treatment recommendations. Future dissemination of these tools will allow more children who have obesity and their families to have access to effective, evidence-based care in diverse communities.

**Trial registration:**

ClinicalTrials.gov identifier: NCT05455190. Registered on 13 July 2022

**Supplementary Information:**

The online version contains supplementary material available at 10.1186/s13012-023-01264-5.

## Contribution to the literature

- While evidence for effective treatment of pediatric obesity has been available for over 15 years, the obesity epidemic continues to worsen, particularly among historically marginalized populations, highlighting a clear gap between evidence and practice.
- Utilizing a novel clinic-community treatment model, this study will close the knowledge-to-action gap by creating accessible and effective web-based implementation tools for the delivery of treatment recommendations.
- Future dissemination of these tools will increase the number of children who have access to effective, evidence-based care and close disparity gaps by offering treatment in diverse community settings.

## Background

Pediatric obesity is a significant public health problem. Nearly 1 in 5 children in the United States (US) have obesity, with racial and ethnic minorities and low-wealth populations disproportionately affected [1]. Obesity during childhood is associated with poorer physical, mental, and social health outcomes and places children at increased risk for obesity during adulthood [2–4]. The United States Preventative Services Task Force (USPSTF) and the American Academy of Pediatrics (AAP) recommend that clinicians screen for obesity beginning as early as 2 years of age using age- and sex-specific body mass index (BMI) [5, 6]. They further recommend that children 6 years and older diagnosed with obesity should be referred to a comprehensive lifestyle intervention that includes nutrition and physical activity components, patient-centered goal setting, and ≥ 26 h of contact over 6–12 months [5, 6]. Evidence shows that this level of treatment leads to measurable and sustainable BMI changes, as well as other benefits to fitness and cardiovascular health [7].

Despite the evidence for effective and safe treatment options, treatment recommendations have largely not been translated into practice. Nationally, rates of nutrition and physical activity counseling in primary care are low, <56% for nutrition and <58% for physical activity, due to a lack of provider time, knowledge, and confidence [8]. Multi-component pediatric weight management programs, often based out of academic medical centers, have emerged to fill this gap; however, they are not universally available, have high attrition rates, and are poorly reimbursed [9]. Furthermore, racial and ethnic communities and low-wealth families often have less access to these tertiary care programs and tend to have lower engagement with clinic-based programs [10–12]. This shows a clear need for additional strategies that provide access to pediatric obesity treatment for diverse populations.

### Fit Together

In order to address the gap between evidence and practice, we designed and tested Fit Together, a pediatric obesity treatment model that meets the AAP and USPSTF recommendations by integrating healthcare and community resources [13, 14]. In the Fit Together model, a partnership is formed between clinical pediatric practices and their local Parks and Recreation organizations. Parks and Recreation organizations represent an ideal community partner with nearly 23,000 parks and 10,000 recreation centers in the 100 largest US cities [15]. Parks and Recreation departments are funded through city or county budgets and are typically located in under-served communities, and the mission includes community wellness [16]. Research has demonstrated that Parks and Recreation programs have the capacity to promote physical activity and cardiovascular health among historically marginalized youth [17, 18]. Furthermore, engaging with Parks and Recreation offers an opportunity for scalability with dissemination through local, state, and national networks.

Within the Fit Together model, clinic partners are responsible for screening and diagnosing children in the clinic, delivering ongoing behavior change counseling to the family using motivational interviewing techniques, treating medical co-morbidities, and ensuring patient safety. Parks and Recreation partners provide recreation center space for nutrition and physical activity programming. Fit Together community sessions are offered 3–5 times per week to provide a high degree of accessibility and flexibility for working families. Sessions are 2 h in duration and provide opportunities to achieve at least 60 min of physical activity through a variety of structured activities and games. Nutrition education sessions are offered once per week. Families are encouraged to attend at least one session per week for 6 months in order to meet obesity treatment guidelines. To facilitate family-level change, parents or caregivers, children, and siblings are invited to participate in all program activities.

A key component of the Fit Together model is the connector [19]. The partners work together to identify and hire a connector, typically an individual who is affiliated with the medical team, but also has experience working in the community (e.g., community health worker, health educator). The connecter is responsible for receiving referrals from pediatric providers and contacting families, leading family orientation sessions, identifying and training staff and volunteers, and delivering on-site activities at the Parks and Recreation facility. The connector also facilitates communication among the clinical and community partners, creating a feedback loop to provide updates on the program.

Research studies have demonstrated that the Fit Together model is feasible to deliver in diverse community settings, acceptable to patients and families, and effective in meeting treatment recommendations and improving child health [13, 20, 21]. Fit Together is modeled after a clinic-community partnership developed between Duke Children’s Healthy Lifestyles pediatric weight management clinic and Durham Parks and Recreation. A mixed methods retrospective analysis of 171 families participating in this program showed that the Fit Together model is able to engage and retain racial and ethnic minorities (31% Black, 44% Hispanic, and 9.4% White) and families with low wealth (69% Medicaid recipients) [20]. Text messaging increased both attendance and engagement with clinic appointments and Fit Together sessions [21]. Results from a randomized controlled trial demonstrated that the program offered 76 possible treatment hours over a 6-month period, exceeding the goal of 26 h [13]. When compared to clinic-only treatment, participation in the Fit Together program was associated with significant improvements in child waist circumference, physical activity, and quality of life [13].

While Fit Together has been shown to be effective, less is known about the factors affecting the implementation of Fit Together in different contexts, which is crucial to inform the dissemination of this treatment model. To begin to address this gap, the Active Recreation through Community-Healthcare Engagement Study (ARCHES) was developed to pilot test the Fit Together model in eight different North Carolina counties. The goal of this pilot study was to understand facilitators and barriers experienced by the clinical and community partners implementing Fit Together and provide evidence of feasibility in delivering the program outside an academic-community partnership. Findings showed that all eight sites were able to develop a partnership, adapt the Fit Together model to their local context, hire and train staff, and deliver Fit Together within 1–12 months (median: 5 months) of forming the partnership, offering on average 76 h (range: 52–93 h) of programming over a 6-month period. Despite reported barriers such as transportation and food insecurity, among participants that attended at least one session (*n*=241), 86% attended more than one session, and 46% achieved the recommended 26 or more hours of treatment [22]. Interviews with partners revealed that the paper-based implementation materials were an important part of developing the partnership and each sites’ Fit Together program [23]. Additional findings included the importance of the connector position, regular meetings for the partners, a streamlined referral system, and a clear endorsement of Fit Together by the referring pediatrician or provider [19].

Building on the ARCHES pilot study, the present study will develop and test an implementation strategy that pairs primary care clinics with municipal Parks and Recreation centers to deliver current treatment recommendations with high fidelity, while allowing for critical adaptions for the local and cultural context. Specifically, this study will design a web-based implementation platform (the Playbook) that will support the development of new partnerships, guide training activities, and provide all the curricular materials needed to implement the Fit Together intervention. The effectiveness of the Playbook as an implementation strategy will be evaluated using an interrupted time series designed and anchored in the Process Redesign Framework. Finally, a dissemination strategy will be developed by applying lessons learned from implementation using the PCORI Dissemination Framework. Evidence for effective treatment of pediatric obesity has been available for over 15 years, while the obesity epidemic has continued to worsen. This project will close the knowledge-to-action gap by creating accessible and effective web-based implementation tools that will allow for the dissemination of evidence-based treatment recommendations in diverse community settings.

## Methods

### Development of the Playbook

We developed a comprehensive implementation package known as the Playbook. The Playbook is a web-based platform that supports the development of new clinic-community partnerships by providing training activities, a formal blueprint for building the partnership and Fit Together program, and all the curricular materials needed to implement the Fit Together intervention. The Playbook utilizes written, video, and audio formats, and includes supporting materials such as checklists, templates for required contracts and forms, and self-assessment components. The development of the Playbook was informed by the paper-based implementation materials and lessons learned in the ARCHES pilot study [19]. Additionally, to ensure that the Playbook balances fidelity to evidence-based treatment recommendations with adaptability to the local context, it was reviewed by several advisory groups with expertise in implementation science, community programming, pediatric obesity treatment, and public policy.

The Playbook contains four units: (1) Fit Together Overview, (2) Learning Modules, (3) Partner Checklist, and (4) Fit Together Connector Guide. A detailed description of each unit is provided in Supplementary Table 1. All partners, including clinical staff, community members, and connectors, are asked to work through the online Playbook modules at their own pace, estimating time to completion of about 10 h.

Briefly, the Fit Together Overview (*Unit 1*) is intended to introduce partners to the clinic-community partnership; describe the shared mission, vision, and goals; and provide the history of the Fit Together program. The Learning Modules (*Unit 2*) provide partners with the necessary training to create a stigma-free environment for children with obesity and provide effective care to empower families throughout the treatment process. Specific trainings include education on weight bias and stigma, motivational interviewing techniques, nutrition and physical activity, and mental health and eating disorders. The Partner Checklist (*Unit 3*) is designed to serve as a blueprint for Fit Together, guiding partners through the steps needed to build the partnership and start their Fit Together program. Activities include guidance on completing all of the necessary contracts and agreements (e.g., shared use or data sharing agreement) between the clinic and community sites, hiring and training the connector, developing the referral process, and planning how to use the community space. Additionally, partners are provided with suggested timing for each step, specific action items to complete each step, and recommended meeting times and topics to foster communication. The Fit Together Connector Guide (*Unit 4*) serves as a resource for the connector to help plan and run the Fit Together community program. The Connector Guide provides guidance on finding staff and volunteers, contacting families and conducting new participant orientation sessions, planning a Fit Together session, and all the logistical considerations associated with running a community program. Finally, in addition to the four units, the Playbook contains a resource section that includes documents and templates needed as part of the Fit Together program (e.g., shared used agreement template, sample connector job description, Fit Together flyers and brochures, and nutrition education curriculum).

#### Other implementation support

In addition to the Playbook, partnerships have access to several other implementation supports.

- Childhood obesity coaches. The AAP Institute for a Healthy Childhood Weight supports a network of experts (“coaches”) available to help local pediatric healthcare providers address obesity in their practices. Leveraging this existing network, Fit Together identified one AAP coach for each site. The coach participates in the training and is available to provide consultation on specific patients who may have medical causes or consequences of obesity.
- Fit Together mobile application. Prior data have demonstrated greater engagement and attendance when families are connected through a mobile device [21]. To help partnerships manage their Fit Together program, and to provide this connectivity directly with families through mobile technology, we have developed an app specifically for Fit Together, in partnership with a third-party vendor, Pattern Health. The Fit Together app provides connectors with a digital tool to track attendance, send messages to families, push out program content, and run a Fit Together incentive program. The Fit Together app also includes the opportunity for children to track steps, set and achieve goals, and earn rewards for positive health behaviors, such as program-branded materials (e.g., water bottles, t-shirts) or fitness equipment.
- Support for sites and families. We have created a public-facing website to provide onboarding and ongoing technical support to local teams, information for new sites who would like to start a Fit Together program in their community, information for families on how to find a Fit Together program, and general nutrition and activity resources for children.

#### Study design

**Fig. 1:**
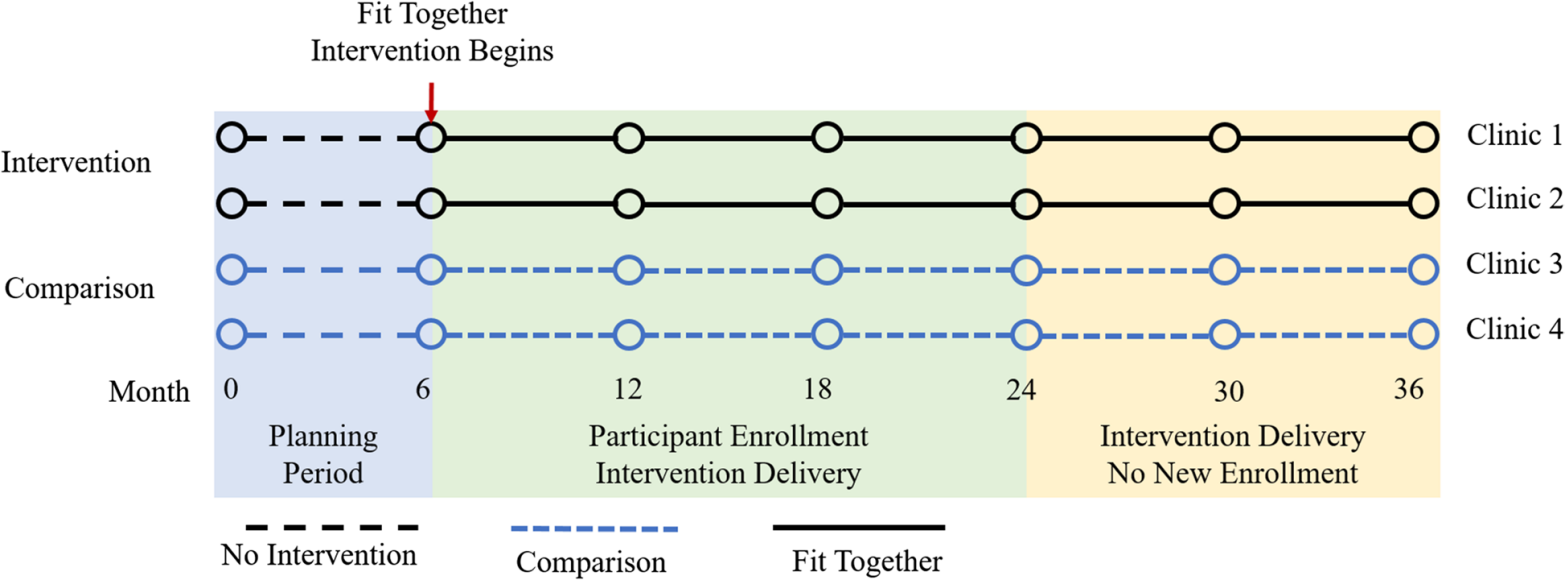
Study flow including planning, participant enrollment, and intervention delivery

A hybrid type II design will be used to evaluate the implementation strategy and to demonstrate the individual-level effectiveness of the Fit Together intervention [24]. Implementation assessment will be guided by The Process Redesign Framework [25]. Embedded within this framework will be a multi-group interrupted time series design for assessment of individual level outcomes. Because both the utility of the implementation strategy and clinical effectiveness are necessary for the development of a comprehensive dissemination and sustainability strategy, they are considered co-primary objectives. Figure 1 shows the study flow. All protocols have been reviewed and approved by the Duke University Health System Institutional Review Board (Pro00106453).

#### Setting, participants, and recruitment

This implementation study will take place in two North Carolina counties, representing large, urban population centers, and areas with high rates of obesity and cardiovascular disease. Within each of these two locations, we have recruited pediatric clinics to deliver the clinical component of Fit Together (*n*=3) and serve as comparisons (*n*=2), and Parks and Recreation facilities (*n*=2) to provide space to deliver the community program.

For implementation measures, we will include all Fit Together partners from each site including pediatric providers and clinic staff, Parks and Recreation leadership and staff, and the connector. Partners will be recruited once each clinic-community partnership begins to use the Playbook. The only inclusion criteria for these individuals are that they are employed by a participating organization, are involved to some degree with the implementation of Fit Together, and can speak English or Spanish.

For effective outcomes, pediatric patients and a caregiver will be recruited through pediatric offices. At well-child visits, pediatric providers will screen children for obesity and refer eligible children to the Fit Together program or the comparison condition using the clinic’s referral process. A member of the research team will contact interested families and enroll them in the study. Then, the connector will contact families to provide additional information about the program and invite them to a new participant orientation session. To be eligible to participate in Fit Together, patients must be between the ages of 6–11 years at the time of enrollment with a BMI ≥ 95th percentile for age and sex, but less than 160% of the 95th percentile. Only children with obesity (not overweight) will be enrolled because the evidence-based model has demonstrated effectiveness only in this group and an important component of the program is that “other children look like me.” Those with a BMI above 160% of the 95th percentile (the most severe obesity) will be referred to a tertiary care weight management program for more intensive treatment. For caregivers to be eligible, they must be 18 years or older, speak English or Spanish, anticipate bringing the child to the program a majority of the time, have no plans to leave the area within the next 12 months, have a smartphone, and be willing to download the apps used in the study for the duration of participation. Although siblings may participate in Fit Together, only the first referred child will be included in the study.

#### Intervention

Sites will implement the Fit Together intervention (described above). Providers in intervention clinics will screen children for obesity, refer them to the community program, and provide medical care appropriate for a child with obesity. Children and families will have the opportunity to participate in the community program for up to 1 year.

#### Comparison

Patients from non-intervention clinics in the same geographic areas will serve as comparisons for individual-level outcomes. Although intervention and comparison clinics may differ somewhat, the interrupted time series design measures trajectory change, and thus provides robust evidence in the context of an implementation study. Qualifying patients who receive primary care at one of the comparison clinics will be given printed materials monthly in the form of a cooking magazine and the local parks and recreation organization newsletter and program guide. Previous studies have demonstrated that this level of intervention is not expected to result in any appreciable weight changes [26].

#### Process redesign framework

The Process Redesign Framework was selected to guide the evaluation of the implementation of Fit Together. This framework provides a way to re-conceptualize how care is delivered and is especially relevant when interventions are complex and implementation is likely to challenge current practices and roles [25]. It was adapted from the Consolidated Framework for Implementation Research (CFIR), retaining the domains and constructs in CFIR, while adding additional domains and constructs relevant to process redesign. Similar to CFIR, the Process Redesign Framework provides a menu of constructs for users to select from to guide the evaluation of implementation. Table 1 shows the domains and relevant constructs from the Process Redesign Framework along with the specific measures that will be used to evaluate the implementation of Fit Together.

**Table 1:** Measurement strategy for implementation study by the component of the Process Redesign Framework

**Constructs**	**Method**
**(1) Int. Chars**	
**What is the intervention designed to achieve? What are the features of the intervention? Who is the intended target group?**	
Intervention description	Validated 20-item survey of staff/provider views of intervention [27]
**(2) Outer setting**	
**What components of the environment will impact the implementation?**	
External policies and incentives	Partner interviews and focus groups at implementation sites
Inter-organizational network structures	
**(3) Inner setting**	
**What components of structure and process within the inner setting will impact the implementation?** [28]	
Implementation Climate	Validated 18-item survey of staff/providers assessing climate [29]
Implementation Leadership	Validated 12-item survey of staff/providers assessing leadership [30]
**(4) Ind./ Team Chars.**	
**What individual or team characteristics of those engaged in the intervention will impact the implementation success and outcomes?**	
Implementer knowledge, attitudes, and beliefs	Clinic and community partner interviews; semi-structured guide adapted from Damschroder [31]
**(5) Process of Implementation**	
**What implementation processes are required to achieve individual- and organizational-level use of the intervention? What roles will individuals and teams carry out?**	
Planning [32]	Meeting notes, document artifacts (e.g., emails), focus groups
Partner engagement [32]	
Executing [32]	
**6) Measures of Implementation**	
**What attributes of the implementation process demonstrate it was carried out well and can be replicated, scaled, and sustained?**	
Acceptability	Validated (4-item scale) survey of staff/providers at intervention sites [33]
Appropriateness	
Feasibility	
Intervention cost	Project administrative data, surveys to assess additional costs
Fidelity	Structured observations of subset of sessions, using SOPLAY [34]
Referrals	Program tracking materials and EHR
Reach	-
a. Reach within the population	Referral, enrollment, and attendance
b.Reach with the organization	Structured interviews with sites
Sustainability	40-item survey of partner covering 8 domains of sustainability [35].
**(7) Outcomes**	
**What specific, measurable outcomes will result from the intervention?**	
Patient experience – Acceptability and Satisfaction	Parent and child satisfaction and acceptability surveys
Health care utilization – Dose/Adherence	EHR records; program participation logs; proportion reaching 26 hours
Cost effects/impact	Project administrative data, surveys to assess additional costs
Unintended consequences	Adverse event reporting
Effectiveness (BMI change)	BMI from clinic records, change in BMI P95 primary, change in BMIz secondary
Effectiveness (Diet and Activity)	Garmin Activity Tracking, diet and physical activity screeners [36–38]
Effectiveness (Quality of Life)	Sizing Them Up [39]

#### Implementation measures

Qualitative measures

Semi-structured interviews and focus groups will be conducted with clinical and community partners 3 months after each site begins implementation of their Fit Together program and will then be conducted annually. Interviews and focus groups will be conducted following the completion of quantitative implementation surveys. Qualitative measures will examine in-depth information about barriers and facilitators to implementation, attitudes, and experiences with the implementation processes, perceptions regarding the intervention adaptability for their local context, and external factors that may affect the implementation of Fit Together (Table 1). Semi-structured guides will be developed for each stage of the project by experts in implementation science using existing interview questions developed to assess CFIR constructs as a guide [31]. Additionally, meeting notes and recordings and document artifacts (e.g., emails) will also be collected.

Quantitative measures

Self-reported surveys completed by partners will be used to assess several constructs within the Process Redesign Framework (Table 1). Survey items are drawn from existing scales including the perceived Characteristics of Intervention Scale, CFIR Inner Setting Scale, Implementation Climate Scale, Implementation Leadership Scale, Acceptability of Intervention Measure, Intervention Appropriateness Measure, Feasibility of Intervention Measure, and the Program Sustainability Assessment Tool [27–30, 33, 35]. Surveys will be completed 3 months following the start of program implementation and will then be conducted annually. To assess the fidelity of the Fit Together community program, structured observations of a subset of program sessions will be conducted throughout the delivery of the community program using the System for Observing Play and Leisure Activity in Youth (SOPLAY). SOPLAY is an observational tool that employs momentary time sampling techniques to systematically document individual (i.e., physical activity levels) and contextual factors (i.e., types of activities) [34]. To assess the reach of Fit Together, referral, enrollment, and attendance data will be collected from the clinics and community sites continuously.

#### Effectiveness measures

Primary outcome

The primary measure of effectiveness will be changed in the child’s BMI at 12 months, defined as the change in BMI relative to the 95th percentile for age and sex (BMIp95). Because all participants will be above the 95th percentile, this measure is a better reflection of change compared to other measures of BMI (i.e., BMI percentile and BMI *z*-score) [40]. Child height, weight, and age at the time of measurement will be extracted from patient medical records at baseline, 6 and 12 months, stored securely in REDCap and used to calculate BMIp95 based on the Centers of Disease Control and Prevention SAS code [41].

Secondary outcomes

Secondary outcomes include children’s objectively measured physical activity and the proportion of children meeting treatment guidelines of ≥26 h of treatment over 6 months. These outcomes will be collected throughout the duration of the study. At baseline, children will receive a Garmin Vivo Fit 4 (Garmin International) to be worn for the duration of the study. Participants will be prompted to synchronize their Garmin device periodically and data will be retrieved from the online platform. Physical activity outcomes include steps and active minutes. To calculate treatment hours, medical visits will be documented from the electronic health record and attendance at the community program will be tracked using the Fit Together app that sites will have access to as part of the implementation support. Additional effectiveness outcomes assessed at baseline, 6 and 12 months include other clinical measures (i.e., blood pressure), dietary behaviors, self-reported physical activity, parent-reported quality of life, social drivers of health, mental health, and assessments of harm [36–39, 42, 43].

#### Power calculation

Power calculations were based on patient-level change in BMIp95. Using data from the National Health and Nutrition Examination Survey, the population mean (SD) for those above the 95th percentile is 117.5 (15.9). Based on the three time points (baseline, 6 months, 12 months), 80% power, and an alpha of 0.05 for a two-sided test, we calculated the required sample size per arm across various plausible standardized differences and a within-subject intra-class correlation coefficient (ICC) of 0.7. Assuming a within-subject ICC of 0.7, we have 80% power to detect a 0.4 standardized decrease (6.98 points) in BMIp95 with 152 subjects retained at 12 months. There are few studies defining clinically meaningful change in BMIp95; however, a 5% reduction in BMIp95 is associated with reductions in cardiovascular disease risk factors [44].

#### Analysis

For implementation analyses, there will be a relatively small sample size from the clinics and community sites; thus, no formal hypothesis testing will be conducted. Quantitative measures will be described using means (SD) for continuous outcomes and frequencies and percentages for categorical outcomes. All interviews and focus groups will be audio-recorded and transcribed verbatim. Transcripts will be analyzed using both deductive and inductive thematic analysis [45].

For participant-level analyses, demographic and clinical characteristics of patients at enrollment will be described by clinic site and by intervention assignment using means (SD) and medians (Q1, Q3) for continuous variables, and frequencies and percentages for categorical variables. Group-level differences will be evaluated as appropriate using Student’s *t* tests, ANOVA, and Kruskal-Wallis testing. The effect of the intervention on clinical outcomes including the patient’s BMIp95 will be evaluated using a clustered interrupted time series approach. The rate and magnitude of change in an individual’s clinical measurement over the study period will be assessed using repeated measures mixed modeling approach. All analyses will include a clustering term for the clinic attended, a variable for assignment to the intervention group, and a time-dependent indicator variable signaling whether a visit occurred in the pre-intervention period versus the post-intervention period. This indicator allows for the detection of differences in the rate of change between the groups once the intervention begins. We will conduct unadjusted intention-to-treat analyses where all patients assigned to an intervention clinic are considered exposed to the intervention. Additional analyses will control for patient-level covariates including patient age, sex, race/ethnicity, and initial BMI.

#### Dissemination strategy

**Fig. 2:**
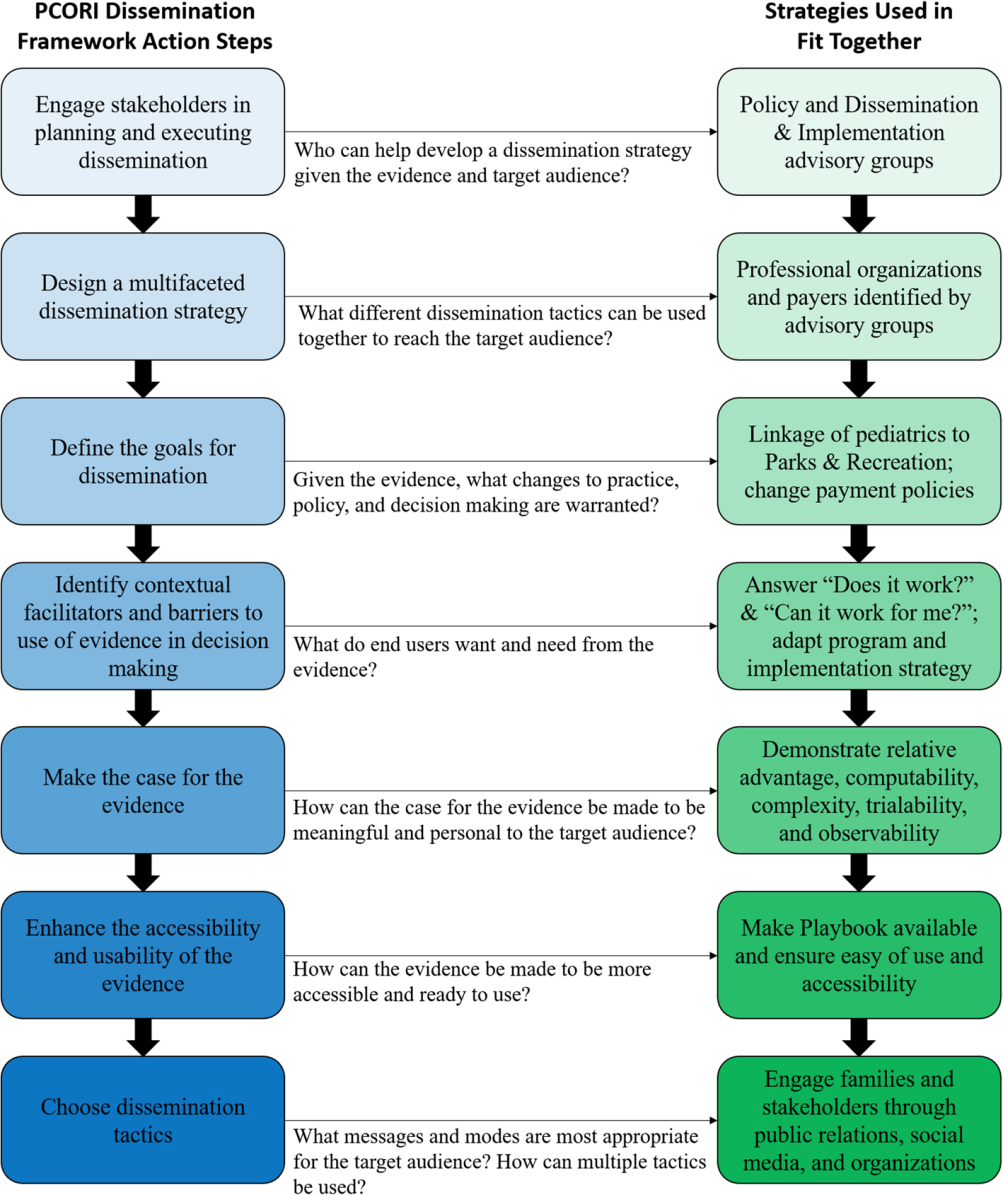
PCORI dissemination framework

Given the limited availability of pediatric obesity treatment, a dissemination plan will be developed to identify the ideal ways to ensure the widespread availability of Fit Together. The PCORI Dissemination Framework will be used to develop and actively implement a dissemination strategy that delivers the Fit Together intervention and implementation strategy through professional and policy channels, community stakeholders (e.g., Parks and Recreation and health care organizations), and academic venues [46]. Each step of the PCORI Dissemination Framework and the strategies used for each step in Fit Together is shown in Fig. 2. Briefly, we will engage partners at each of our sites as well as policy and dissemination and implementation advisory groups throughout the study to ensure a plan for sustainability (step 1). The advisory groups will assist in the development of the remaining strategies including the identification of appropriate targets and needs for dissemination, refining the goals for dissemination, and using study findings to tailor components of the implementation strategy for other communities (steps 2-4). Using Rogers’s Diffusion of Innovation of Theory, we will create reports appropriate for all stakeholders demonstrating each component of the model: relative advantage (advantage over other treatments), compatibility (compatibility with current processes), complexity (Playbook provides step by step guide), trialability (ability to “try out” program before making long-term commitment), and observability (outcomes can be easily observed through tools in the Playbook) (step 5) [47]. Following the completion of the study, the Playbook will be made available to the public as a web-based resource and shared with relevant organizations such as the National Parks and Recreation Association and the American Academy of Pediatrics (step 6). Finally, dissemination tactics will be adapted and expanded upon based on findings from the implementation study in order to facilitate widespread dissemination (step 7).

## Discussion

The status quo for pediatric obesity treatment research has been to develop an intervention and then test the effectiveness using a randomized, comparative effectiveness, or quasi-experimental design [7]. While these studies have been important to establish the evidence base, little guidance exists on how to implement interventions in real-world settings [48]. The field of implementation science provides the necessary tools to move effective interventions into practice, yet there has been limited research on the application of these methods in the context of pediatric obesity treatment [49]. This study represents a substantive departure from the status quo, as we move away from testing new weight-loss interventions, and move towards implementation and dissemination of existing evidence to real-world settings for the promotion of healthy behavior change.

Other departures from the status quo include a focus on historically marginalized populations, an emphasis on sustainability, and expanding the focus on outcomes beyond BMI. Prior studies have not enrolled high numbers of racial and ethnic minorities and children from low-wealth families, despite the fact these populations are at risk of experiencing adverse outcomes related to obesity [50]. Fit Together intentionally engages racial and ethnic minorities and low-wealth groups, as the referring clinics serve a population of diverse children and over 85% are insured by Medicaid. Sustainability is also an important component of Fit Together, as it is emphasized throughout the process of planning and implementing Fit Together, whereas many other studies rely on research funding to support the innovation. In the current study, the Playbook will be made freely available at the conclusion of the study to support the continued sustainability of Fit Together at each site and remove barriers to future uptake. We have also engaged stakeholders within the National Recreation and Park Association and AAP in order to leverage existing resources to support sustainability. Additionally, our high-level policy advisory board, which includes national leaders in healthcare reform, will focus on identifying opportunities for reimbursement under innovative and emerging value-based care directives. Finally, the current study will move away from defining “effectiveness” based on BMI change. BMI change is often prioritized at the expense of objective measures of behavior change (e.g. physical activity) that precede BMI reduction and are known to reflect concrete health improvements.

This study has several potential challenges. First, it is possible that we will face difficulties enrolling patients in the community program through the referral process. Our previous work suggests a high degree of variability in referral success from clinic to clinic, with anywhere from 9-36% of those referred actually attending the community program [19]. To plan for the possibility of low enrollment, we will conduct an ongoing assessment of the referral procedure and implement additional implementation support as needed. Additionally, by working with health systems with multiple pediatric practice locations, we are able to add additional clinics as needed. A second challenge is potential connector turnover, which we experienced in our pilot study and impeded implementation in some settings. We hope to limit connector turnover by using community health workers who are already embedded within the clinic infrastructure. If we do experience turnover, the web-based Playbook will serve as a standardized training tool to quickly and efficiently bring new connectors on board. Finally, study participant attrition is a concern. We have developed a number of strategies to reduce attrition including frequent contact with families using text message check-ins, incentives for participation and prizes for engagement, and the use of primary and secondary outcomes that do not require in-person study visits.

## Conclusion

The goal of Fit Together is to provide all children with access to safe and effective pediatric obesity treatment. To achieve this goal, the current study will provide evidence-based implementation tools that support clinicians and community partners in delivering effective pediatric obesity treatment recommendations. Future dissemination of these tools will increase the number of children who have access to effective, evidence-based care and close disparity gaps by offering treatment in diverse community settings.

## Supplementary Information

**Additional file 1:** **Table 1. **Description of the units contained within the Playbook implementation support tool.

## Data Availability

Not applicable.

## Abbreviations

AAP: American Academy of Pediatrics
ARCHES: Active Recreation through Community-Healthcare Engagement Study
BMI: Body mass index
BMIp95: BMI relative to the 95th percentile for age and sex
CFIR: Consolidated Framework for Implementation Research
ICC: Intraclass correlation coefficient
PCORI: Patient-Centered Outcomes Research Institute
SD: Standard deviation
SOPLAY: System for Observing Play and Leisure Activity in Youth
US: United States
