# Supplementary material for: Translating knowledge into action for child obesity treatment in partnership with Parks and Recreation: study protocol for a hybrid type II trial
Source: Implement Sci. 2023 Feb 24;18:6. doi: 10.1186/s13012-023-01264-5 (PMC9951422; doi:10.1186/s13012-023-01264-5)
Supplement: Supplementary file 1 — Additional file 1: Table 1. Description of the units contained within the Playbook implementation support tool. [file 13012_2023_1264_MOESM1_ESM.docx]

| **Supplementary Table 1.** Description of the units contained within the Playbook implementation support tool | | |
| --- | --- | --- |
| **Unit** | **Topics Covered/Action Items** | **Resources Provided** |
| **Unit 1: Fit Together Overview** | | |
| Welcome to the Fit Together Playbook | - Overview of what to expect while going through the Playbook including suggested timing |  |
| The Clinic Community Partnership | - Role of each partner - Fit Together vision, mission and goals |  |
| Fit Together: How it Works | - Overview of the Fit Together including how and why it works for children with obesity |  |
| The First Fit Together: Bull City Fit | - Introduction to how Fit Together was created and the evidence of the effectiveness of the program |  |
| **Unit 2: Learning Modules ^a^** | | |
| Childhood Obesity Overview | - Definition and prevalence of obesity - How to support children with obesity | Links and suggestions for further information |
| Nutrition 101 for Children with Obesity | - The link between what we eat and are our health; - Supporting healthy eating at any weight; - Tips for healthy eating on a budget - Common eating patterns of young children | Links and suggestions for further information |
| Adaptive Activity 101 for Children with Obesity | - Benefits of being active - Physical activity guidelines for youth - Tips for physical activity promotion among children - Encouraging positive attitudes around physical activity - Considerations unique to children with obesity | Links and suggestions for further information |
| Mental Health and Eating Disorders | - Defining mental health and its role in healthy living - Unique mental health needs of children with obesity - Understanding what an eating disorder is and how to treat | Links and suggestions for further information |
| Weight Bias and Stigma | - Understanding weight bias and stigma and the consequences - What can you do to combat weight bias and stigma | Links and suggestions for further information |
| Motivational Interviewing | - Defining motivational interviewing - Importance of motivational interviewing techniques - Elements of motivational interviewing - Putting it all together: the four process of motivational interviewing | Links and suggestions for further information |
| **Unit 3: Partner Checklist** | | |
| Build the Team | Topics   - Defining the roles of each team member (the clinical champion, recreation champion, child obesity coach, and connector)   Action Items (*6-12 months before launch date*)   - Identify the Clinical Champion, the Recreation Champion, and the Child Obesity Coach. - Schedule regular meetings to work through steps before program launch. - Review Logo Design Templates - Create a program name! | - Logo design samples - Fit Together overview presentation |
| Complete Contracts and Agreements | Topics   - Overview of the types of agreements that may be needed (shared use agreement, collaboration agreement, data sharing agreement)   Action Items (*begin at least 12 months before launch date*)   - Review the “Shared Use Agreement Template” and “Shared Use Agreement Fact Sheets” - Review the "Scope of Work Example" - Complete all necessary agreements between healthcare and recreation center entity | - Scope of work example - Shared use agreement fact sheet - Shared use agreement template - Contracts and agreements supplementary checklist |
| Hire and Train the Connector | Topics   - Process for hiring connector - Qualities to look for in a connector   Action Items (*2-3 months before launch date*)   - Review the “Sample Connector Job Description” - Review “Scope of Work Example” - Post position and interview - Once the connector is hired, participate in orientation and training activities | - Sample connector job description |
| Develop Referral Process (clinical partners) | Topics   - Tips for developing the referral process - Importance of provider during the referral process   Action Items (*1-3 months before launch date*)   - Review "Child obesity management and referral flowchart" document and make any edits specific to your practice - Download the "Referral Template" and customize for your practice - Engage providers and clinic staff to decide the optimal workflow for filling out referrals and getting them to the Connector | - Childhood obesity management and referral flowsheet - Referral form template |
| Plan the Recreation Spaces (recreation partners) | Topics   - Considerations for facility, space, and equipment (number of people, space, times and days; equipment needed; prizes/incentives for participants; materials needed for specific activities and the nutrition curriculum) - Overview of Fit Together session content   Action Items (*1-3 months before launch date*)   - Review relevant materials in Connector Guide (Unit 4), particularly:   - "Getting to Know the People and Spaces"   - "Planning a Fit Together Session": Fitness First Fifteens, Group Games, Healthy Tastes curriculum - Work with Connector to complete the Equipment and Inventory Planning Tool - Plan where Fit Together will be held based on space usage throughout the year. - Consider local awards your recreation center may be eligible to receive - Develop materials advertising the program |  |
| Gather for a Pre-Launch Kickoff Meeting | Topics   - Goal of the kickoff meeting   Action Item (*2-4 weeks before launch*)   - Conduct kickoff meeting |  |
| **Unit 4: Fit Together Connector Guide** | | |
| Fit Together Connector Overview | Topics   - Fit Together mission, vision and goals   Action Item   - Watch videos to learn about the Fit Together program and connector role |  |
| Rules of Play | Topics   - Review Rules of Play: Play Hard, Play Fair, Play Safe, Play as a Team - Consequences if rules are not followed - Additional rules and expectations   Action Items   - Create your own Rules of Play poster - Create a rules and expectations handout to share with families at orientation | - Rules of Play poster - Rules and expectations handout |
| Getting to know the People and Spaces | Topics   - Determining resources and purchasing supplies - Safety planning - Building a relationship with the clinical and recreation center staff - Setting up a feedback loop with the clinic and community partners   Action Items   - Complete the equipment and inventory planning tool - Use the sample safety protocol to create a safety plan with your partners | - Equipment and inventory planning tool - Sample safety protocol |
| Setting up the Referral System and Contacting Families | Topics   - Typical referral process and eligibility criteria - Contacting and talking with families - Promoting Fit Together in clinic - Lessons learned about referrals   Action Items   - Create a 1-page Fit Together orientation flyer and share with clinical partners - Develop a Fit Together family information brochure and share with your clinical partners - Write your own Fit Together welcome letter to send to new participants | - Family brochure template - Orientation flyer template - Welcome letter |
| Volunteers and Staff | Topics   - Recruiting and selecting the right volunteers - Paid staff - Clearances and trainings - Volunteer orientation and training; volunteer scheduling - Helping volunteers and staff feel pride in their work   Action Items   - Recruit volunteers using the volunteer interest scripts - Train volunteers using the Fit Together volunteer orientation sample presentation - Set up a volunteer spreadsheet using the Fit Together volunteer information tracker | - Volunteer orientation sample presentation - Volunteer interest script - Volunteer information tracker |
| Using Pattern Health | Topics   - Using Pattern Health for your Fit Together program - Who should have Pattern Health on their phone - Attendance and sending message to participants - Staying healthy between Fit Together sessions - Fit Together points and prizes   Action Items   - Read the Pattern Health mobile app guide - Complete training on the Pattern Health mobile app with the Duke team | - Guide (update) |
| New Participant Orientation | Topics   - Planning your new participant orientation - Membership agreement and waiver - The new participant orientation welcome talk and facility tour   Action Items   - Create your new participant orientation welcome talk using the new participant orientation template presentation - Talk with recreation center to reserve space/times/materials for the new participant orientation - Prepare your membership agreement and waiver | - New participant orientation presentation - Sample membership agreement and waiver |
| Planning a Fit Together Session | Topics   - Fit Together session logistics - Fit Together session content - Unstructured play and warm-ups - Healthy Tastes nutrition lessons - Closing and prizes - What do caregivers do during a Fit Together session? - Troubleshooting some common issues during a session   Action Items   - Read and become familiar with the Fit Together fitness first 15s, groups games, and healthy tastes lessons guides - Print and laminate the 24 healthy tastes posters and store in a safe place - Use the Fit Together session planning tool to put together a mock session and submit to Duke team for feedback - Attend a Fit Together session in another community, if possible | - Session planning tool - Fitness first 15s - Group games - Taste testing curriculum - Nutrition education posters |
| Fit Together Celebrations | Topics   - Fit Together Kid of the Week - Graduation - Volunteer recognition   Action Items   - Decide on how you will choose and honor the Kid of the Week - Discuss the graduation plans with your recreation center partners to get their ideas, reserve dates/times |  |
| (a) All Learning Modules in Unit 2 contain an informational video with an expert in the topic area, 1-2 pages of informational content, and quizzes to assess comprehension of the subject matter | | |
